# COVID-19 – Treiber für die Digitalisierung des Einzelhandels? Eine Untersuchung in vier Mittelstädten

**DOI:** 10.1007/s00548-022-00824-z

**Published:** 2023-01-03

**Authors:** Christopher Herb, Carla Friedrich, Cordula Neiberger

**Affiliations:** Geographisches Institut, Lehr- und Forschungsgebiet Wirtschaftsgeographie der Dienstleistungen, RWTH Aachen, Wüllnerstr. 5b, 52062 Aachen, Deutschland

**Keywords:** Geographische Handelsforschung, Inhabergeführter Einzelhandel, Digitalisierung, Nordrhein-Westfalen, Mittelstädte, Geographic trade research, Owner-led retail, Digitalization, North Rhine-Westphalian, Medium-sized towns

## Abstract

Die COVID-19-Pandemie hat nicht nur die Gesellschaft, sondern auch die Wirtschaft, darunter speziell den Einzelhandel, vor große Herausforderungen gestellt. Während Onlinehändler von der Schließung lokaler Geschäfte profitierten, wurde dem stationären Einzelhandel plötzlich die Haupteinnahmequelle entzogen. Dieser befindet sich seit Jahrzehnten in einem Strukturwandel, der sich zuletzt durch die umfassende gesellschaftliche und wirtschaftliche Digitalisierung neuen Herausforderungen stellen musste. Unser Beitrag beschäftigt sich mit der Frage, inwieweit Handelsunternehmen sich gezwungen sahen, eine verstärkte Digitalisierung, hier untersucht als Präsenz im Internet, durch die Coronalockdowns vorzunehmen. Wir betrachten unterschiedliche Onlinestrategien, die von einer kompletten Inaktivität bis hin zu einer umfassenden Onlinestrategie (Onlineshops, unterstützt durch Social-Media-Aktivitäten) reichen. Um die Entwicklung in den letzten Jahren sowie über drei COVID-19-Lockdowns sichtbar zu machen, stellt die Untersuchung einen Vergleich zwischen zwei Erhebungszeitpunkten (2017/18 und 2021) anhand vierer Mittelstädte in Nordrhein-Westfalen (Baesweiler, Düren, Eschweiler, Heinsberg) dar. Es zeigt sich, dass der Digitalisierungsprozess grundsätzlich voranschreitet, wenngleich das Ausmaß der digitalen Aktivitäten stark von der jeweiligen Organisationsform abhängt. Zudem lassen sich auch Unterschiede hinsichtlich der Größe der Mittelstädte erkennen.

## Abstract (en)

The COVID-19 pandemic has posed major challenges not only to society but also to business, including the retail sector in particular. Although online retailers benefited from lockdowns and the closure of local stores, brick-and-mortar retailers were cut off from their main source of income overnight. The latter has been undergoing structural change for decades and has recently had to face new challenges as a result of extensive social and economic digitalization. Our article deals with the question of the extent to which retailers felt compelled to undertake increased digitization as a consequence of the COVID-19 lockdowns. We consider different online strategies, ranging from complete inactivity to a comprehensive online strategy (online shops, supported by social media activity). In order to visualize the development over the last years as well as over three COVID-19 lockdowns, the study presents a comparison of two survey dates (2017/2018 and 2021) based on four medium-sized towns in North Rhine-Westphalia (Baesweiler, Düren, Eschweiler, Heinsberg). It can be seen that the digitalization process is progressing in general, although the extent of digital activities is strongly dependent on the respective form of organization. In addition, differences can also be seen with regard to the size of the medium-sized towns.

## Einführung

Der Einzelhandel befindet sich, getrieben von Innovationen und veränderten gesellschaftlichen Rahmenbedingungen in einem steten Entwicklungsprozess. Während frühere Neuerungen „Selbstbedienung“ oder „Shoppingcenter“ hießen, ist seit einigen Jahren die umfassende gesellschaftliche und ökonomische Digitalisierung ein starker Treiber. Bislang wird diese Entwicklung speziell vom stationären Facheinzelhandel weniger als Chance, sondern vielmehr als Herausforderung wahrgenommen. Auf der einen Seite ist ein stetiges Wachstum des Internethandels zu erkennen, auf der anderen Seite sind Modernisierungen aufgrund fehlender Ressourcen und einem großen Beharrungsvermögen, aber auch wegen ungeklärter Generationenfragen bislang häufig ausgeblieben. Während Filialisten den Anforderungen der Digitalisierung i. d. R. folgen können, schaffen inhabergeführte Fachhandelsunternehmen oftmals eine Anpassung nicht: So mussten innerhalb von zehn Jahren (2010–2019) 34.500 Fachhandelsunternehmen schließen, in der gleichen Zeit entstanden knapp 1000 Fachmarkt- bzw. Filialunternehmen (HDE/IFH Köln 2020, S. 20).

**Abb. 1:**
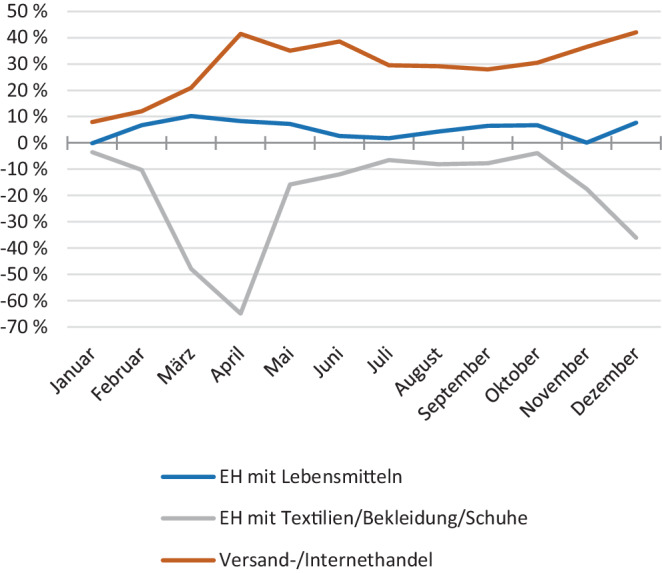
Veränderungen im Umsatz zum Vorjahresmonat im Einzelhandel 2020 in NRW (verändert nach IT NRW 2021b)

Mit Beginn der COVID-19-Pandemie wurden Einzelhandelsunternehmen nun zusätzlich vor große Herausforderungen gestellt. Insbesondere die drei bundesweit angeordneten Lockdownphasen und die damit einhergehenden Maßnahmen setzten die stationären Unternehmen stark unter Druck. Lediglich der Erwerb von Produkten des täglichen Bedarfs (z. B. Lebensmittel und tlw. Heimwerkerbedarf) war während dieser Phasen möglich. Folglich war der Versand‑/Internethandel zum Teil die einzige Möglichkeit, Produkte zu verkaufen bzw. zu erwerben. Daher ist es nicht verwunderlich, dass sich Pandemie und Lockdowns sehr unterschiedlich auf den Einzelhandel ausgewirkt haben. Besonders deutlich werden diese Unterschiede im Vergleich zwischen den drei Branchen „Einzelhandel mit Lebensmitteln“, „Einzelhandel mit Textilien/Bekleidung/Schuhe“ und „Versand‑/Internethandel“, wie Abb. 1 für Nordrhein-Westfalen (NRW) verdeutlicht (IT NRW 2021b).

Während der drei Lockdownphasen zwischen März 2020 und Mai 2021 stellten digitale Aktivitäten (neben Telefon und Fax) die einzige Möglichkeit für den Einzelhandel dar, mit Konsumenten zu interagieren und Umsätze zu erzielen. Die Verbraucher reagierten entsprechend. So stieg der Onlineumsatz in Deutschland von 2019 zu 2020 um 23 % und zu 2021 nochmals um 19 %. Damit wurden im Jahr 2021 86,7 Mrd. € online umgesetzt, was einem Anteil am Gesamteinzelhandel von 14,7 % entspricht (HDE/IFH 2022). Eingekauft wurde insbesondere über Marktplattformen, aber auch die Onlineshops des stationären Einzelhandels konnten Umsatzzuwächse verzeichnen (HDE/IFH 2022).

Damit stellt sich die Frage, ob und wie die Unternehmen gerade des inhabergeführten Einzelhandels auf diese Herausforderung mit einer verstärkten digitalen Tätigkeit (Webseiten, Aktivitäten in Sozialen Medien, Google My Business-Einträge oder Onlineshops) reagierten. Die vorliegende Untersuchung präsentiert eine Vollerhebung der Entwicklung aller Einzelhandelsunternehmen in vier Städten NRWs und gibt damit einen größeren Überblick über das Digitalisierungsverhalten von Handelsunternehmen.

## Methodik

Die vorliegende Untersuchung greift auf Daten aus den Jahren 2017/18 zurück, in denen der Stand der Digitalisierung in sechs Städten des IHK-Bezirks Aachen untersucht wurde. Diese Untersuchungen wurden nach Beendigung des dritten Lockdowns 2021 in vier der Städte wiederholt, um die Auswirkungen des COVID-19-Lockdowns auf die Digitalisierungsbemühungen der Handelsunternehmen nachvollziehen zu können.

Zu beiden Untersuchungszeiträumen wurde dieselbe Methodik herangezogen: zum einen eine Begehung der Geschäftsstraßen der zentralen Versorgungsbereiche, zum anderen eine „desktop research“, in dem Organisationform, Art des Onlineauftritts (kein Onlineauftritt, Webseite oder Onlineshop) und Tätigkeiten in Sozialen Medien (Facebook und Instagram) erhoben wurden.

Aus den Kategorien Onlinepräsenz und Auftritt in Sozialen Medien wurden die Onlinestrategien der Unternehmen abgeleitet, die von „inaktiv“ bis zu „umfassende Onlinestrategie“ reichen (Tab. 1, Neiberger und Kubon 2018).

**Tab. 1:** Onlinestrategien nach der gewählten Onlinepräsenz und der Aktivität in sozialen Netzwerken. (Verändert nach Friedrich et al. 2022, S. 66; basiert auf Neiberger und Kubon 2018, S. 23)

Onlinepräsenz	Aktivität in sozialen Netzwerken	
	Nein	Ja
Kein Onlineauftritt	Inaktiv	Kundenpflege
Homepage	Informationsstrategie	Informationsstrategie mit Kundenpflege
Onlineshop	Verkaufsstrategie	Umfassende Onlinestrategie

## Baesweiler, Düren, Eschweiler und Heinsberg – vier Mittelstädte im Vergleich

**Abb. 2:**
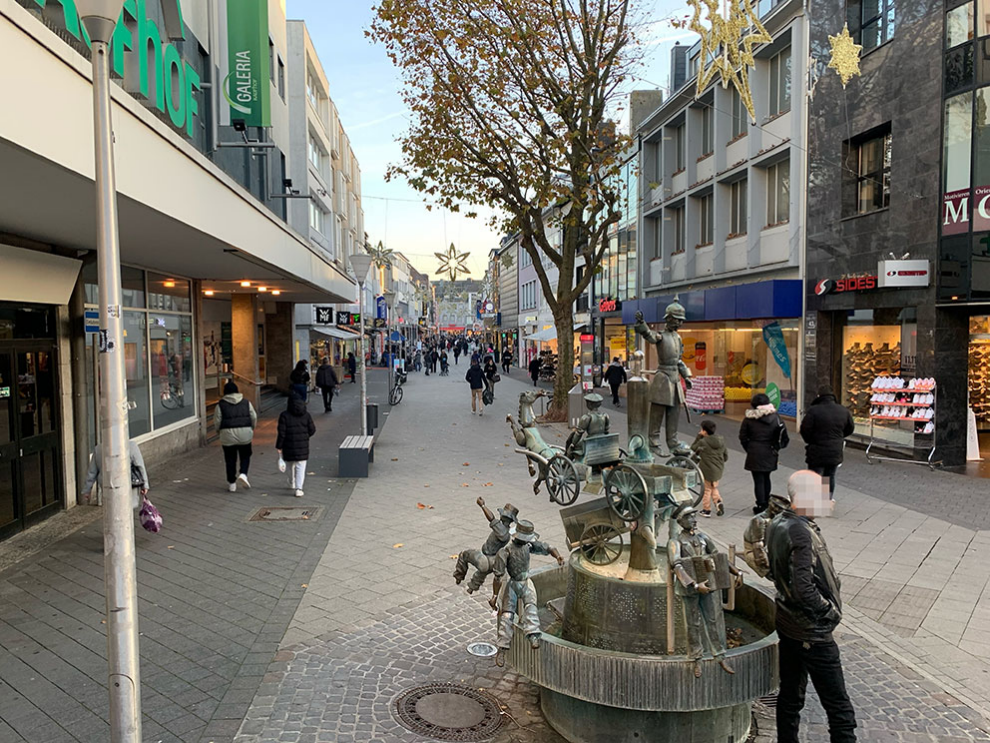
Innenstadt Düren. (Foto: Neiberger)

Nach einer Studie des BBSR (2017, S. 60) wird der Handel in Großstädten auch in Zukunft von einer positiven Entwicklung geprägt sein. Demgegenüber stellen speziell Klein- und Mittelstädte die „Sorgenkinder“ im Einzelhandelsbereich dar, sind sie doch „*in besonderem Maße von den Folgen des Strukturwandels im Einzelhandel betroffen – ihnen droht die Verödung der Handelslandschaft*“ (BearingPoint/IIHD 2015, S. 8). Während Kleinstädte als beinahe sichere Verlierer gelten, kann die Zukunft von Mittelstädten noch nicht abschließend bewertet werden (BearingPoint/IIHD 2015, S. 10 ff.; Neiberger et al. 2020, S. 206). Die Entwicklungen sind dabei neben verschiedenen Strukturdaten wie Bevölkerungsentwicklung, Kaufkraft und Einzugsbereiche immer auch von der Einbettung der Unternehmen in ihren sozioökonomischen Kontext abhängig. Für den vorliegenden Beitrag wurden vier Mittelstädte in NRW herangezogen, die sich hinsichtlich verschiedener Kennzahlen zum Teil durchaus enorm unterscheiden (siehe Tab. 2, Abb. 2).

**Tab. 2:** Kennzahlen der vier Städte im Jahr 2021. (IHK Aachen 2021a, b, c; IT NRW 2021a, c, d, e, f)

	Düren	Eschweiler	Heinsberg	Baesweiler
Nähe zum nächsten Oberzentrum (Aachen)	28 km	14 km	32 km	16 km
Bevölkerung (30.06.2021)	91.350	56.141	42.692	27.313
Sozialversicherungspflichtig Beschäftigte (31.12.2019)	44.940	19.748	17.746	5941
Einzelhandelsumsatz (2020)	608,0 Mio. €	335,7 Mio. €	268,1 Mio. €	105,4 Mio. €
Einzelhandelszentralitätskennziffer (2020)	134,9	114,4	121,5	77,7
Kaufkraftindex (2020)	91,1 (5973 €/P.)	93,1 (6289 €/P.)	93,2 (6303 €/P.)	89,9 (6019 €/P.)

Tab. 3 bietet einen Überblick über die Anzahl der kartierten Geschäfte in den vier Vergleichsstädten. Es zeigt sich, dass der inhabergeführte Einzelhandel in allen vier Kommunen die Mehrheit des stationären Einzelhandels stellt. Besonders ersichtlich ist dies in der kleinsten Stadt, Baesweiler, in der der Anteil bei ca. 77 % liegt. Die Städte Eschweiler und Heinsberg weisen mit je 61 % den geringsten Anteil auf.

**Tab. 3:** Untersuchungssample der Geschäfte (2021) in Baesweiler, Düren, Eschweiler und Heinsberg. (Eigene Erhebung)

	Düren		Eschweiler		Heinsberg		Baesweiler	
	Anzahl	%	Anzahl	%	Anzahl	%	Anzahl	%
Inhabergeführt	302	65	123	61	68	61	65	77
Andere Organisationsformen	162	35	78	39	44	39	20	23
Gesamt	464	100	201	100	112	100	85	100

## Onlinepräsenzen von Einzelhandelsunternehmen in Mittelstädten

**Abb. 3:**
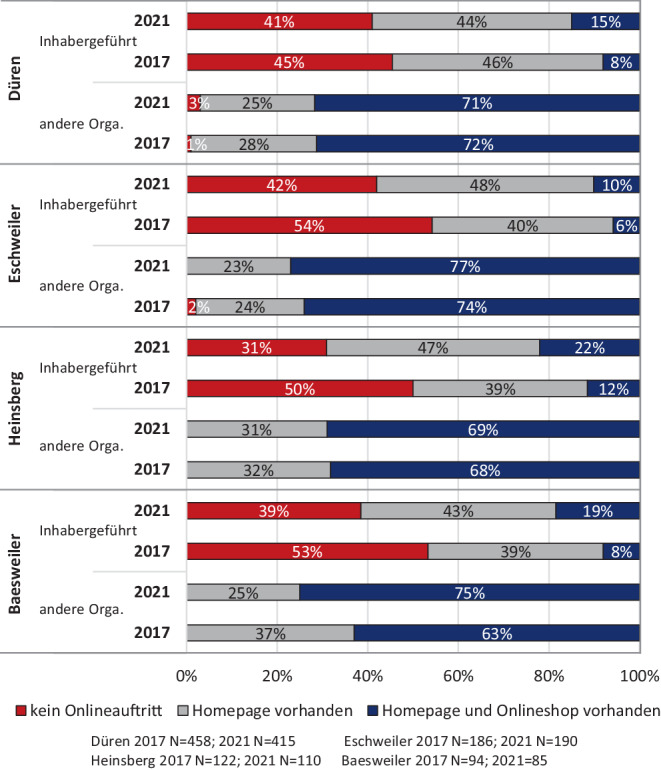
Onlinepräsenz der Einzelhandelsunternehmen nach Städten und Organisationsform. (Eigene Erhebung)

Eine Präsenz im Internet ist mittlerweile für einen Großteil der Unternehmen eine Selbstverständlichkeit und auch die Kunden erwarten eine solche. Nichtsdestotrotz gibt es immer noch einen durchaus großen Anteil von kleinen Einzelhandelsunternehmen ohne jeglichen Onlineauftritt (Abb. 3). Im Jahr 2021 sind es außer in Heinsberg, wo ca. 30 % der Händler weder eine Webseite noch einen Onlineshop aufweisen, in allen anderen Städten sogar ca. 40 %.

Der Anteil der inaktiven Unternehmen des inhabergeführten Handels war jedoch im Jahr 2017 noch höher. Der Vergleich der Daten mit 2017/18 zeigt in allen vier Städten eine Zunahme sowohl der Webseitenpräsenzen als auch von Onlineshops bei gleichzeitigem Rückgang der Inaktivität, wenngleich in unterschiedlichem Maße. In Düren ging die Inaktivität um lediglich vier Prozentpunkte zurück. In den beiden kleineren Städten ist dagegen eine deutlichere Zunahme von Onlineaktivitäten zu verzeichnen, dabei handelt es sich sowohl um die Einrichtungen von Webseiten als auch von Onlineshops.

Einen niedrigschwelligen Einstieg in eine Onlinepräsenz bietet das Tool Google My Business (google my business 2022). Jedoch verfügen eher Unternehmen mit Webseite oder Onlineshop über einen entsprechenden Eintrag, weniger die inaktiven Unternehmen. Google My Business dient wohl eher als Ergänzung zu bereits bestehenden Onlinestrategien bei digital aktiven Einzelhandelsunternehmen und weniger als Ersatz für sonstige Digitalisierungsmaßnahmen.

## Aktivitäten in Sozialen Medien

*Soziale Netzwerke* haben sich in den letzten Jahren in der Kommunikation mit Konsumenten als wichtige Säule etabliert. Direktes Feedback, gezielte Ansprache und eine erhöhte Bekanntheit sind als Vorteile zu nennen. Zudem ist die Installation und das Betreiben eines Social-Media-Auftrittes mit deutlich weniger Know-how, Budget und Zeitressourcen verbunden als der Aufbau einer Webseite oder eines Onlineshops.

**Abb. 4:**
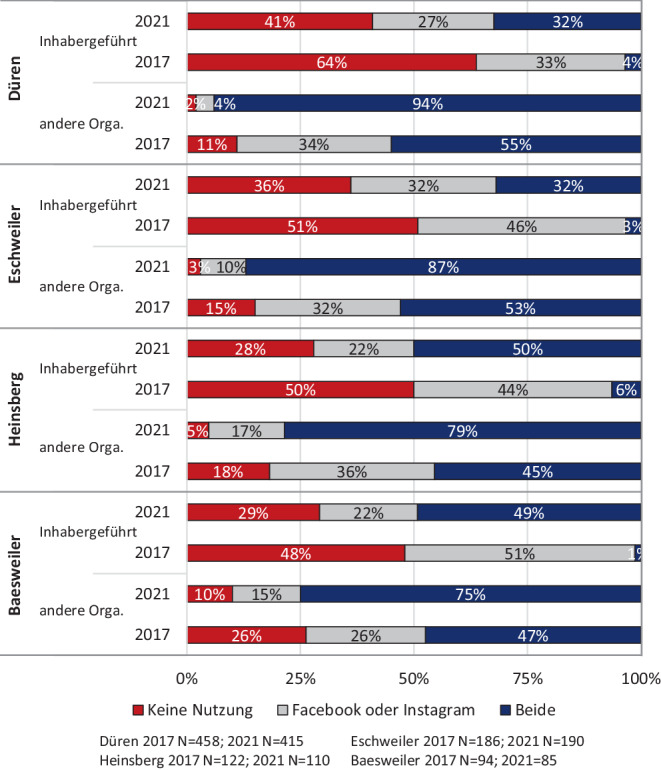
Aktivitäten in Sozialen Medien nach Städten und Organisationsformen. (Eigene Erhebung)

Trotzdem lassen sich auch bei der Nutzung Sozialer Medien deutliche Unterschiede zwischen dem inhabergeführten Einzelhandel und anderen Organisationsformen (Filialunternehmen, Franchiseunternehmen, Shops von Herstellern) feststellen, zudem aber auch zwischen den Städten (vgl. Abb. 4). So sind in Düren 41 % der inhabergeführten Unternehmen nicht in Sozialen Netzwerken aktiv, während es in den kleineren Städten Heinsberg und Baesweiler nur 28 bzw. 29 % sind. Gerade in diesen zwei Städten lässt sich jedoch eine starke Zunahme der Aktivitäten seit 2017/18 konstatieren. Dies lässt die Vermutung aufkommen, dass der direkte und persönliche Kundenkontakt in kleineren Städten eine größere Bedeutung innehat.

## Welche Onlinestrategien verfolgen Einzelhandelsunternehmen?

**Abb. 5:**
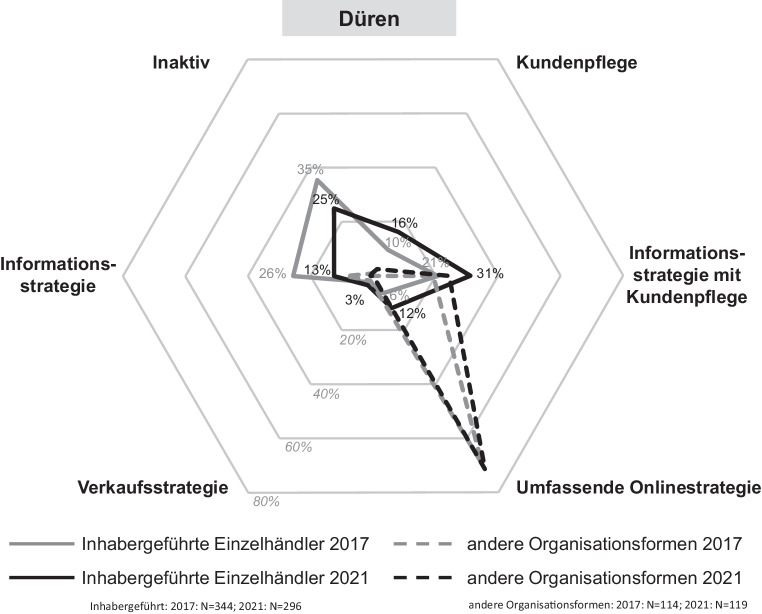
Onlinestrategien der Einzelhandelsunternehmen in Düren. (Eigene Erhebung)

**Abb. 6:**
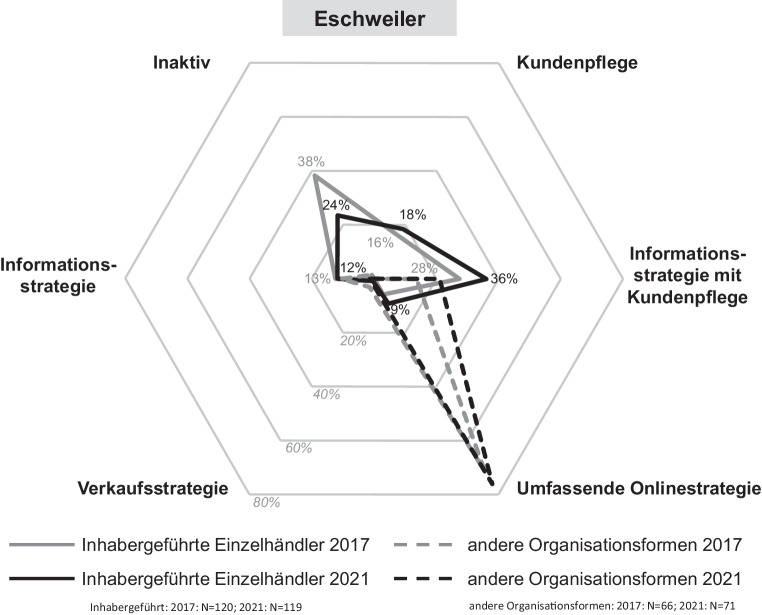
Onlinestrategien der Einzelhandelsunternehmen in Eschweiler. (Eigene Erhebung)

Die Digitalisierungsaktivitäten von Einzelhandelsunternehmen werden durch verschiedene Onlinestrategien gekennzeichnet. Je nach der individuellen Umsetzung in den Bereichen Onlinepräsenz und Aktivität in sozialen Netzwerken reichen diese von einer umfassenden bis zu einer inaktiven Onlinestrategie. In den Abb. 5, 6, 7 und 8 sind die Onlinestrategien der vier Städte – unterschieden nach dem inhabergeführten Einzelhandel und denen der anderen Organisationsformen als Benchmark – in deren zeitlicher Entwicklung dargestellt.

**Abb. 7:**
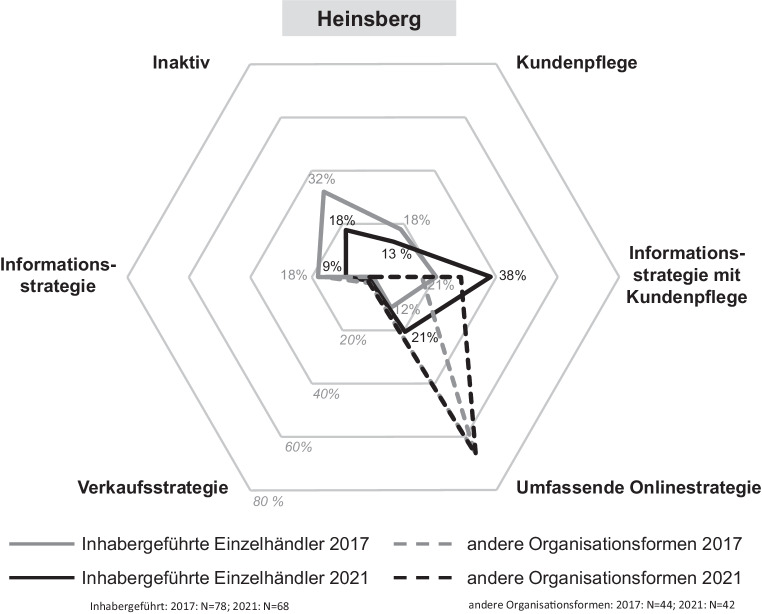
Onlinestrategien der Einzelhandelsunternehmen in Heinsberg. (Eigene Erhebung)

**Abb. 8:**
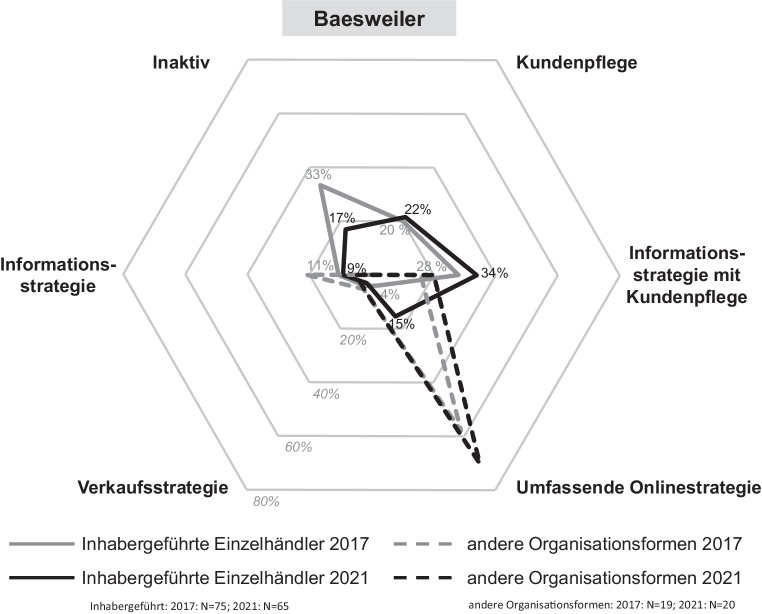
Onlinestrategien der Einzelhandelsunternehmen in Baesweiler. (Eigene Erhebung)

In *Düren* verfolgt der Großteil der inhabergeführten Einzelhandelsunternehmen (31 %) die Informationsstrategie mit Kundenpflege (Webseite und mindestens ein Social-Media-Auftritt), gefolgt von der Inaktivität, die immer noch ein Viertel der Unternehmen ausmacht. Eine umfassende Onlinestrategie setzen lediglich 12 % der Handelsunternehmen um (Onlineshop und Social-Media-Auftritt). Im Gegensatz dazu zeigt sich bei den anderen Organisationsformen ein komplett anderes Bild. Mit 71 % verfolgt die Mehrheit der Unternehmen eine umfassende Onlinestrategie, also sowohl Onlineshop als auch Aktivitäten in sozialen Netzwerken. Darüber hinaus betreiben 24 % eine Informationsstrategie mit Kundenpflege. Die restlichen Onlinestrategien sind nur zu sehr geringen Anteilen vertreten.

Die grau dargestellten Linien ermöglichen einen Vergleich mit dem Jahr 2017. Die Inaktivität der inhabergeführten Einzelhandelsunternehmen hat um 10 Prozentpunkte und die Informationsstrategie um 13 Prozentpunkte abgenommen. Dafür ist die Kundenpflege um 6 Prozentpunkte und die Informationsstrategie mit Kundenpflege um 10 Prozentpunkte gestiegen. Auch die umfassende Onlinestrategie hat ein Wachstum von 6 % auf 12 % vorzuweisen. Verallgemeinert kann die Aussage getroffen werden, dass es eine Verschiebung hin zu Strategien mit Social Media gegeben hat. Die Veränderungen der anderen Organisationsformen sind auf geringerem Niveau.

Bei Betrachtung der zweitgrößten Stadt *Eschweiler* zeigt sich ein sehr ähnliches Bild. Ebenso stellt sich die Situation in der kleinsten untersuchten Stadt *Baesweiler* dar. Hier ist lediglich hervorzuheben, dass nur noch 17 % des inhabergeführten Einzelhandels online vollkommen inaktiv ist, was den kleinsten Wert aller untersuchten Städte bedeutet.

Die Onlinestrategien in *Heinsberg* lassen dagegen Unterschiede erkennen. Hervorzuheben ist der vergleichsweise hohe Anteil der inhabergeführten Handelsunternehmen mit einer umfassenden Onlinestrategie. Diese lag bereits im Jahr 2017 mit 12 % über dem heutigen Anteil in Düren und Eschweiler. Im Jahr 2021 ist der Anteil noch einmal deutlich auf 21 % angestiegen. Dies ist der höchste Wert im Städtevergleich. Die vollkommene Inaktivität der inhabergeführten Einzelhandelsunternehmen erreicht mit ca. 18 % einen geringeren Wert als Düren und Eschweiler. Aber auch hier ist die Informationsstrategie am stärksten ausgeprägt.

## Fazit

Zusammenfassend kann festgehalten werden, dass sich der Digitalisierungsgrad von Einzelhandelsunternehmen in der Zeitspanne zwischen 2017/18 und 2021 hin zu einer digitaleren Sichtbarkeit erhöht hat. Nichtsdestoweniger lassen sich über alle vier Untersuchungsstädte enorme Unterschiede in der Entwicklung zwischen inhabergeführten Unternehmen und anderen Organisationsformen, wie Filialisten und Franchisenehmer, erkennen. Letztere verfolgen hauptsächlich eine umfassende Onlinestrategie, eine völlige Inaktivität ist nur in Ausnahmefällen vorhanden. Demgegenüber vertrauen inhabergeführte Einzelhandelsunternehmen verschiedenen Onlinestrategien, wobei die Informationsstrategie mit Kundenpflege in allen Städten den größten Anteil einnimmt. Dies ist sicherlich nicht nur Ausdruck fehlender Ressourcen, sondern durchaus auch eine strategische Entscheidung vieler kleiner Händler mit eher austauschbarem Sortiment, die den Preisdruck im Internet vermeiden möchten (und müssen).

Erfreulicherweise hat sich die Inaktivität in allen Kommunen deutlich reduziert, wenngleich sie immer noch auf je mindestens 17 % aller inhabergeführten Einzelhandelsunternehmen zutrifft. Ein besonders hohes Wachstum ist im Bereich der Sozialen Medien zu verzeichnen. Aufgrund der geringen finanziellen und organisatorischen Einstiegshürden stellen Soziale Medien offenbar ein gutes Mittel dar, online sichtbar zu werden.

Der Vergleich zeigt jedoch auch, dass die Entwicklung in der Digitalisierung stufenweise vonstattengeht. So werden Webseiten oder Soziale Medien eingerichtet, selten aber beides gleichzeitig. Häufig sind Aktivitäten in Sozialen Medien ein erster Einstieg, weil die Einstiegshürden hier am geringsten sind, denn diese können durch affine Mitarbeiterinnen oder Mitarbeiter und Familienangehörige geleistet werden. Auch hier ist zu beobachten, dass zunächst nur in ein Tool eingestiegen wird, wohl um Erfahrungen zu sammeln.

Auch ein externer Schock, wie die Lockdowns der COVID-19-Pandemie, verändern dieses Vorgehen der schrittweisen Aneignung von Know-how und Entwicklung der Strategie nicht. Einzelhandelsunternehmen eröffnen in den seltensten Fällen direkt Onlineshops, wenn sie zuvor vollkommen inaktiv waren. Dies ist auch in der Notwendigkeit einer entsprechenden Vorbereitungszeit begründet, die während der COVID-19-Pandemie nicht gegeben war. Zudem war zu keinem Zeitpunkt klar, wie lange ein Lockdown dauern würde und wie oft ein solcher wiederholt werden müsste. Es konnte also nie abgeschätzt werden, inwieweit sich eine solche Investition überhaupt lohnen würde. Nur Unternehmen, die sich sowieso mit dem Gedanken der Einrichtung eines Onlineshops getragen haben, haben diesen nun auch umgesetzt.

Die auch nach den Lockdowns immer noch hohe Anzahl an Unternehmen des inhabergeführten Einzelhandels, die gar keine digitale Aktivität zeigen, lässt auf strategische Entscheidungen der Unternehmer schließen. Diese unterscheiden sich in ihrer Persönlichkeit, ihrer Onlineaffinität, ihrer Zielsetzung und den vorhandenen Ressourcen zum Teil erheblich.

Die Lockdowns der COVID-19-Pandemie führten somit nicht zu einer weitreichenden, umfassenden und schnellen Digitalisierung des stationären inhabergeführten Einzelhandels. Diese waren eben nur eine (kurze) Episode in einer langen Geschäftigkeit, die immer wieder durch externe Einflüsse Herausforderungen ausgesetzt ist. Der seit vielen Jahren andauernde Strukturwandel des Einzelhandels wurde dadurch zwar verschärft, ein Wandel in der Strategie der Unternehmen haben die Lockdowns aber eher selten hervorgerufen.

Obwohl sich die Untersuchungsstädte hinsichtlich Größe und Bedeutung des Einzelhandels durchaus stark unterscheiden, wurden keine wesentlichen Unterschiede in den Digitalisierungsaktivitäten der ansässigen Unternehmen festgestellt. Lediglich die erhöhte Aktivität in Sozialen Medien in den kleineren Städten lassen hier auf eine stärkere Verbindung zu Stammkunden schließen, die eine nun digitale Kundenpflege möglich und notwendig erscheinen ließen.

## References

[CR8] IHK Aachen (2021a) Gemeindeprofile Kreis Düren. https://www.aachen.ihk.de/blueprint/servlet/resource/blob/3332120/3e00601c24fdf2b364a84607672ecd96/gemeindeprofile-kreis-dueren-data.pdf. Zugegriffen: 16. Nov. 2021

[CR9] IHK Aachen (2021b) Gemeindeprofile Kreis Heinsberg. https://www.aachen.ihk.de/blueprint/servlet/resource/blob/3332148/7e7902bc5fe35dba359c85ef609056a9/gemeindeprofile-kreis-heinsberg-data.pdf. Zugegriffen: 16. Nov. 2021

[CR10] IHK Aachen (2021c) Gemeindeprofile Städteregion Aachen. https://www.aachen.ihk.de/blueprint/servlet/resource/blob/3332152/f8659db5c126ef8e698a3f8ba7d78c02/gemeindeprofile-staedteregion-aachen-data.pdf. Zugegriffen: 16. Nov. 2021

[CR1] BBSR (2017) Online-Handel – Mögliche räumliche Auswirkungen auf Innenstädte, Stadtteil- und Ortszentren. https://www.bbsr.bund.de/BBSR/DE/veroeffentlichungen/bbsr-online/2017/bbsronline-08-2017-dl.pdf;jsessionid=653912949FF5C700C1A3AD5EF73D7DA2.live11313?__blob=publicationFile&v=1. Zugegriffen: 22. Juni 2022

[CR2] BearingPoint/IIHD (2015) Strukturwandel im deutschen Einzelhandel. Warum gerade Klein- und Mittelstädte von den Folgen des Strukturwandels im Einzelhandel besonders betroffen sind. https://www.bdu.de/media/32083/manke-studie-8.pdf. Zugegriffen: 22. Juni 2022

[CR3] Friedrich C, Herb C, Neiberger C (2022) Soziale Medien, Webseiten oder Onlineshops? (Digitale) Reaktionen des Einzelhandels auf die Covid-19-Krise. In: Appel A, Hardaker S (Hrsg) Innenstädte, Einzelhandel und Corona in Deutschland. Würzburg University Press, Würzburg, S 61–86

[CR5] Google My Business (2022) Einfach online gefunden werden – mit Ihrem kostenlosen Unternehmensprofil auf Google. https://www.google.com/intl/de_de/business/. Zugegriffen: 22. Juni 2022

[CR6] HDE/IFH Köln (2020) Standortmonitor 2020. https://einzelhandel.de/component/attachments/download/10376. Zugegriffen: 22. Juni 2022

[CR7] HDE/IFH Köln (2022) Online-Monitor 2022. https://einzelhandel.de/index.php?option=com_attachments&task=download&id=10659. Zugegriffen: 22. Juni 2022

[CR11] IT NRW (2021a) Bevölkerung in Nordrhein-Westfalen. https://www.it.nrw/statistik/eckdaten/bevoelkerung-nach-gemeinden-93051. Zugegriffen: 16. Nov. 2021

[CR12] IT NRW (2021b) Gewinner und Verlierer des Corona-Lockdowns: Gegenläufige Umsatzentwicklung im NRW-Einzelhandel im Jahr 2020 in einzelnen Wirtschaftszweigen. https://www.it.nrw/gewinner-und-verlierer-des-corona-lockdowns-gegenlaeufige-umsatzentwicklung-im-nrw-einzelhandel-im. Zugegriffen: 8. Nov. 2021

[CR13] IT NRW (2021c) Kommunalprofil Baesweiler, Stadt. https://www.it.nrw/sites/default/files/kommunalprofile/l05334008.pdf. Zugegriffen: 16. Nov. 2021

[CR14] IT NRW (2021d) Kommunalprofil Düren, Stadt. https://www.it.nrw/sites/default/files/kommunalprofile/l05358008.pdf. Zugegriffen: 16. Nov. 2021

[CR15] IT NRW (2021e) Kommunalprofil Eschweiler, Stadt. https://www.it.nrw/sites/default/files/kommunalprofile/l05334012.pdf. Zugegriffen: 16. Nov. 2021

[CR16] IT NRW (2021f) Kommunalprofil Heinsberg, Stadt. https://www.it.nrw/sites/default/files/kommunalprofile/l05370016.pdf. Zugegriffen: 16. Nov. 2021

[CR18] Neiberger C, Kubon J (2018) Onlinemonitor. Onlinestrategien des stationären Einzelhandels in den Kommunen Aachen, Düren, Euskirchen, Eschweiler, Heinsberg und Baesweiler. https://www.dlgeo.rwth-aachen.de/global/show_document.asp?id=aaaaaaaaaxcixuk&download=1. Zugegriffen: 10. Aug. 2022

[CR19] Neiberger C, Mensing M, Kubon J (2020) Geographische Handelsforschung im Zeitalter der Digitalisierung: Eine Bestandsaufnahme. Z Wirtschgeogr 64(4):197–210

